# Supplementary material for: Healthcare professionals’ perspectives on barriers and facilitators to implementing a warning signs intervention for older rural-dwelling medical patients at risk for hospital readmission
Source: PLoS One. 2025 May 5;20(5):e0322138. doi: 10.1371/journal.pone.0322138 (PMC12052142; doi:10.1371/journal.pone.0322138)
Supplement: Attachment — Submitted filename: PLos_One_HCP Hierarchies_Response to Reviewers_26feb2025.docx [file pone.0322138.s002.docx]

**Response to Reviewer Comments Received Dec. 17, 2024**

**Manuscript ID:** PONE-D-24-37531

**Title:** Healthcare professionals' perspectives on barriers and facilitators to implementing a warning signs intervention for older rural-dwelling medical patients at risk for hospital readmission

| **Comment** | **Response** |
| --- | --- |
| **Academic Editor Comments** |  |
| Ethics Statement: The ethics approval is mentioned on page 5, and the statement indicates that written informed consent was obtained from all participants. It aligns well with PLOS ONE's requirement for transparency about ethical considerations. However, the statement could be more explicit about the nature of the informed consent process (i.e., whether it was written or verbal). | We have provided more detail about our written informed consent process including that (1) a research associate provided potential participants with the consent form and the opportunity to meet by telephone to review the study and what participation entails, (2) participants were offered the opportunity to ask questions vie telephone and/or email, and (3) consenting participants sent their signed consent form to the research associate electronically. |
| Data Availability: The manuscript confirms that all data are fully available without restriction, as per PLOS ONE's data sharing policy. However, specific details on the repository or data accessibility may be needed. As stated on page 7, it confirms that "All relevant data are within the manuscript and its Supporting Information files."  Ensure that no conflicting statements appear within the manuscript, such as stating that data is available on request elsewhere. | All relevant data are within the manuscript (Table 3 lists all participant quotations). Consequently, details on a repository are not required.  We have ensured that no conflicting statements appear within the manuscript related to data availability. |
| Methodology section: The methodology on pages 13-17, detailing the qualitative descriptive design, is well laid out, but additional explanation might be needed regarding how the sample size of 42 participants was determined. This should be explained to ensure it is consistent with typical qualitative research expectations (heterogeneity of the respondents), as qualitative sample sizes are often determined by saturation rather than pre-set numbers. | We explain that sampling for focus groups and interviews was conducted until it was determined that informational saturation was reached (i.e., when further data collection became redundant) |
| Result section: The presentation of results, including tables on pages 19-20, is thorough. However, additional clarifications might be necessary in terms of how the data was analyzed and how different strata (nurses, physicians, allied health professionals) were compared. Ensure that the methods section clearly explains the data coding and analysis processes, especially the use of NVivo 12 and qualitative content analysis, and the role of the two coders (pages 15-17). | We now elaborate that the data were examined for patterns in healthcare professionals’ narratives based on their strata using role-ordered and conceptually clustered matrices and analytic memoing. Specifically, we used matrices to map out each stratum of healthcare professionals’ perspectives on the barriers and facilitators, which were summarized within each stratum, and within and across settings (hospital and community).  We also clarify that the coding process entailed having each coder independently develop a list of potential codes and then comparing them for divergence and overlap.  Lastly, we explain that coding was facilitated by NVivo 12, which was used to consolidate transcripts, perform first- and second-cycle coding by applying codes to segments of text, and collate narrative quotes to support reporting. |
| Abstract: The abstract is clear, but it could benefit from a stronger emphasis on the specific outcomes or findings of the study rather than focusing predominantly on the background and aim. The key findings could be briefly summarized to help the reader understand the significance of the barriers and facilitators identified. | In addition to stating the key findings, we now state: Seven of these categories converged across healthcare professional strata. However, the reasons why different healthcare professional strata perceived the categories as important, and the ways in which they saw them functioning as barriers and facilitators, varied. Our findings shed light on barriers and facilitators that should be considered to ensure successful implementation of the intervention in rural communities. |
| Results Table (Table 1 on page 19): While the table is informative, a brief explanation in the text regarding the presence or absence of certain barriers and facilitators across different strata could enhance clarity. For example, there could be a deeper dive into why certain barriers were not identified by specific strata (e.g., "Risks & Liabilities" not being a concern for nurses). | We now indicate that unlike some allied health care professionals, nurses see the warning signs intervention to be within their scope of practice and that this could explain why nurses did not identify risks and liabilities as a barrier. |
| Citations: There are some references to literature in the introduction (pages 9-12), but more recent sources may be required to ensure the study is grounded in the latest research, particularly concerning rural healthcare and warning signs interventions. | We have updated the literature in the introduction except for one citation (HQO, 2012). We contacted Health Quality Ontario (HQO) to see if they have done any further research and were told that they have not. In addition, we had a librarian conduct a search and found no more recent evidence. Given the scarcity of rural research in this area (warning signs interventions in rural communities), and to give context, we believe it is important to include this citation, and elected to do so, even though the source is dated. |
| Grammar and Formatting: Some minor formatting inconsistencies are noticeable, such as the inconsistent use of "HCPs" (Healthcare Professionals) and the way quotes are integrated into the text. Ensure that all acronyms are defined on their first use and that the flow of written text remains coherent. | We have carefully reviewed the manuscript for any formatting inconsistencies and we define all acronyms. |
| Conclusion section: The manuscript could strengthen its conclusion by linking the barriers and facilitators to practical recommendations for policy implementation or future research, especially since the findings could have a direct impact on improving transitional care in rural settings. | We did not have a conclusion section but have emphasized more clearly the policy implications in the section labeled implications for policy. |
| **Journal Requirements** |  |
| When submitting your revision, we need you to address these additional requirements.  1. Please ensure that your manuscript meets PLOS ONE's style requirements, including those for file naming. The PLOS ONE style templates can be found at  [https://journals.plos.org/plosone/s/file?id=wjVg/PLOSOne_formatting_sample_main_body.pdf](https://track.editorialmanager.com/CL0/https:%2F%2Fjournals.plos.org%2Fplosone%2Fs%2Ffile%3Fid=wjVg%2FPLOSOne_formatting_sample_main_body.pdf/1/010f0193d71eaa96-e9005c19-f641-4e9c-bc7f-015a17d120b2-000000/zYwNUDkUxjJA0ZtBE7Tn90qe4fsbzqX7MWrTpOgwep0=189) and  [https://journals.plos.org/plosone/s/file?id=ba62/PLOSOne_formatting_sample_title_authors_affiliations.pdf](https://track.editorialmanager.com/CL0/https:%2F%2Fjournals.plos.org%2Fplosone%2Fs%2Ffile%3Fid=ba62%2FPLOSOne_formatting_sample_title_authors_affiliations.pdf/1/010f0193d71eaa96-e9005c19-f641-4e9c-bc7f-015a17d120b2-000000/LGSy0Z1P8yqM0hG8XIkGamQfP-77bGjCsAxIuh-9N9A=189) | We have ensured that the manuscript meets style requirements. |
| Thank you for stating the following financial disclosure:  “This work is supported by the Canadian Institutes of Health Research, Funding Reference Number 163072, awarded to M.T.F. The Canadian Institutes of Health Research had no role in the design, data collection and analysis, decision to publish, or preparation of the manuscript. [https://www.cihr-irsc.gc.ca/e/193.html”](https://www.cihr-irsc.gc.ca/e/193.html)  Please state what role the funders took in the study.  If the funders had no role, please state: "The funders had no role in study design, data collection and analysis, decision to publish, or preparation of the manuscript."  If this statement is not correct you must amend it as needed.  Please include this amended Role of Funder statement in your cover letter; we will change the online submission form on your behalf. | We have amended the financial disclosure statement in the cover letter as requested.  In the manuscript and in our cover letter, we now state that the funders had no role in study design, data collection and analysis, decision to publish, or preparation of the manuscript. |
| 3. We note that your Data Availability Statement is currently as follows: “All relevant data are within the manuscript and its Supporting Information files.”  Please confirm at this time whether or not your submission contains all raw data required to replicate the results of your study. Authors must share the “minimal data set” for their submission. PLOS defines the minimal data set to consist of the data required to replicate all study findings reported in the article, as well as related metadata and methods ([https://journals.plos.org/plosone/s/data-availability#loc-minimal-data-set-definition](https://track.editorialmanager.com/CL0/https:%2F%2Fjournals.plos.org%2Fplosone%2Fs%2Fdata-availability%23loc-minimal-data-set-definition/1/010f0193d71eaa96-e9005c19-f641-4e9c-bc7f-015a17d120b2-000000/gE66TS2X0zOdO9d9tj4aAJSM6wDVMgrHQUaobu7B3kk=189)).  For example, authors should submit the following data:  - The values behind the means, standard deviations and other measures reported;  - The values used to build graphs;  - The points extracted from images for analysis.  Authors do not need to submit their entire data set if only a portion of the data was used in the reported study.  If your submission does not contain these data, please either upload them as Supporting Information files or deposit them to a stable, public repository and provide us with the relevant URLs, DOIs, or accession numbers. For a list of recommended repositories, please see [https://journals.plos.org/plosone/s/recommended-repositories](https://track.editorialmanager.com/CL0/https:%2F%2Fjournals.plos.org%2Fplosone%2Fs%2Frecommended-repositories/1/010f0193d71eaa96-e9005c19-f641-4e9c-bc7f-015a17d120b2-000000/-ZUP8nhVtECUPFfDl4NTFAod0m_8VZfPWi3fuCsavwg=189).  If there are ethical or legal restrictions on sharing a de-identified data set, please explain them in detail (e.g., data contain potentially sensitive information, data are owned by a third-party organization, etc.) and who has imposed them (e.g., an ethics committee). Please also provide contact information for a data access committee, ethics committee, or other institutional body to which data requests may be sent. If data are owned by a third party, please indicate how others may request data access. | All relevant data are within the manuscript. |
| **Reviewer 1** |  |
| The introduction highlights the unique challenges faced by the elderly as they transition from hospital to home care and thus provide valuable contribution to the literature by identifying the barriers and facilitators to implementation of health care interventions in this regard in rural settings. There is need to emphasize the uniqueness of the research and its applicability across other similar settings in order to fully understand the study's importance and extent of the research. | To more strongly emphasize the study’s uniqueness, we now explain that prior research has not explored healthcare professionals’ perspectives on barriers and facilitators to implementing a warning signs intervention, particularly in rural communities where such interventions are urgently needed to prevent avoidable patient deterioration and unnecessary readmissions. We also emphasize that such interventions are urgently needed to prevent avoidable patient deterioration in health and unnecessary hospital readmissions.  To clarify the applicability of our research to other similar settings, we now explain in the section on sample and setting, that rural dwellers in Ontario face challenges such as limited access to care due to a lack of health care providers, which are common in rural areas across North America. Rural Ontario thus constitutes an ideal research recruitment setting. |
| The study could benefit from a more thorough literature review of existing research to compare findings and applicability. | We have provided a more thorough review of existing research in both the introduction and discussion sections. |
| Qualitative methodology was an adequate scientific methodology for this study. However, how were the study participants selected (was it a purposive or convenience sampling), this will assist in assessing the sample representativeness. | We now explain that participants were purposefully recruited based on the eligibility criteria listed in the section on “Setting and Sample”. |
| The study would be better presented with a more detailed explanation of its sampling, data collection and analysis methodology for example inter-coder reliability. Also, while themes were provided clearly, the coding methods and thematic analysis should be provided explicitly. | See response above for sampling and response to editor’s comments for data analysis. |
| The results are presented clearly but are mostly descriptive and lack adequate analysis especially regarding interrelating themes. These should be explored with more depth and presented rather than repeating all of the quotes. | Our data analysis approach is in line with our qualitative descriptive design which aims to minimize data transformation in favour of presenting the data in participants’ own terms. Our list of quotes in the table is to provide sufficient evidence to substantiate our descriptive findings. |
| To support validity, some results could benefit from statistical/evidence-based justifications. | The study employed a qualitative descriptive design to examine healthcare professionals’ perspectives on potential barriers and facilitators to providing a warning signs intervention. We did not employ quantitative measures; consequently, we are unable to provide statistical justifications. However, we have compared our findings more thoroughly to evidence in the literature in cases where evidence was available. |
| Discussions identifies key barriers like health awareness and staffing constraints but should be more robust to include policy recommendations and scalability of the interventions. The discussions should provide some comparisons of results from other similar studies to highlight convergent or divergent themes and suggest some broader implications. Practical and concise policy and practice recommendations, grounded in evidence would increase the impact of this study. | We now provide more comparisons of our results to other similar studies and we provide more recommendations such as policy makers can use our findings to prioritize inter-sectoral communication by linking community and hospital electronic medical records. |
